# In Situ Sprayed Biotherapeutic Gel Containing Stable Microbial Communities for Efficient Anti‐Infection Treatment

**DOI:** 10.1002/advs.202205480

**Published:** 2022-12-07

**Authors:** Jian‐Hua Yan, Di‐Wei Zheng, Hui‐Yun Gu, Yun‐Jian Yu, Jin‐Yue Zeng, Qi‐Wen Chen, Ai‐Xi Yu, Xian‐Zheng Zhang

**Affiliations:** ^1^ Key Laboratory of Biomedical Polymers of Ministry of Education & Department of Chemistry Wuhan University Wuhan 430072 P. R. China; ^2^ Department of Orthopedic Trauma and Microsurgery Zhongnan Hospital of Wuhan University Wuhan 430071 P. R. China

**Keywords:** anti‐infection, gels, kombucha, microbes, multidrug‐resistance

## Abstract

Systematic administration of antibiotics to treat infections often leads to the rapid evolution and spread of multidrug‐resistant bacteria. Here, an in situ‐formed biotherapeutic gel that controls multidrug‐resistant bacterial infections and accelerates wound healing is reported. This biotherapeutic gel is constructed by incorporating stable microbial communities (kombucha) capable of producing antimicrobial substances and organic acids into thermosensitive Pluronic F127 (polyethylene‐polypropylene glycol) solutions. Furthermore, it is found that the stable microbial communities‐based biotherapeutic gel possesses a broad antimicrobial spectrum and strong antibacterial effects in diverse pathogenic bacteria‐derived xenograft infection models, as well as in patient‐derived multidrug‐resistant bacterial xenograft infection models. The biotherapeutic gel system considerably outperforms the commercial broad‐spectrum antibacterial gel (0.1% polyaminopropyl biguanide) in pathogen removal and infected wound healing. Collectively, this biotherapeutic strategy of exploiting stable symbiotic consortiums to repel pathogens provides a paradigm for developing efficient antibacterial biomaterials and overcomes the failure of antibiotics to treat multidrug‐resistant bacterial infections.

## Graphical abstract

In situ sprayed biotherapeutic gel containing stable microbial communities is constructed to control multidrug‐resistant bacterial infections and accelerate wound healing. The active antimicrobial substances and organic acids secreted by the microbial community have remarkable abilities to self‐stabilize and antagonize exogenous pathogens, while avoiding bacteria‐induced systemic inflammation.

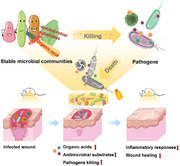

## 1 Introduction

Healthy skin is in direct contact with the external environment, where a variety of microbial communities, including bacteria, fungi, and viruses, coexist. These microbes have significant impacts on the maturation and homeostasis of the immune system.^[^ ^1^, ^2^, ^3^ ^]^ Once the skin is damaged, however, the open, moist, and nutritious wound provides an exclusive environment for pathogen colonization. Uncontrolled pathogen clearance and barrier restoration result in epidermal barrier deficiencies and wound infections.^[^ ^4^, ^5^, ^6^ ^]^ To combat infections, patients generally receive systemic broad‐spectrum antibiotic treatments. Unfortunately, dynamically tricky pathogens constantly evolve sophisticated mechanisms to resist invariable antibiotics, which drives the rapid evolution and spread of multidrug‐resistant bacteria.^[^ ^7^, ^8^, ^9^ ^]^ Accordingly, it is extremely desirable to develop an adaptable strategy to manage drug‐resistant bacterial infections and promote tissue regeneration.

Microbes are highly gregarious species and their social intelligence can modulate a range of behaviors that are important for fitness, including the ability to remodel and subsequently evolve in response to environmental cues.^[^ ^10^, ^11^, ^12^ ^]^ In particular, certain beneficial bacteria can create a unique local microenvironment by secreting large amounts of metabolites and antimicrobial agents, that are suited for their survival but inhibit the growth of competing microorganisms.^[^ ^2^, ^13^ ^]^ *Lactobacilli*, for example, have been successfully utilized to treat urinary tract infections by restoring the natural vaginal flora.^[^ ^14^ ^]^ Furthermore, yeasts and filamentous fungi can coexist in a shared environment and exhibit both synergistic and antagonistic interactions.^[^ ^10^ ^]^ However, in the contest of bacteria‐based biotherapeutics, studies have demonstrated that the uncontrolled growth of bacteria might cause unsatisfactory treatment efficacy and systemic inflammation.^[^ ^15^, ^16^ ^]^

Kombucha is known as a kind of fermentation product, carried out by a microbial consortium including yeasts, *Komagataeibacter*, and often *Lactobacillus* genera in sugared tea liquid.^[^ ^17^, ^18^ ^]^ During fermentation, yeasts hydrolyze polysaccharides into monosaccharides by invertase actions and subsequently bacteria remove waste products through alcohol metabolization. The first stages of interconnected biochemical reactions are dominated by osmotolerant microorganisms and later by acid‐tolerant species.^[^ ^19^ ^]^ The interactions likely represent a symbiotic relationship between bacteria and yeasts, resulting in a stable co‐culture system.^[^ ^20^ ^]^ Although there have been many works on the use of kombucha for antibacterial purposes, most of the studies are limited to in vitro antibacterial assays, with a single means of characterization and a lack of suitable dosage forms.^[^ ^21^, ^22^, ^23^ ^]^Together, we speculated that such an ingenious symbiont possessed the ability to repel foreign bacteria and maintain the original homeostasis, which could serve as a living biotherapeutic to control infection with decreased systemic inflammation.

**Scheme 1:**
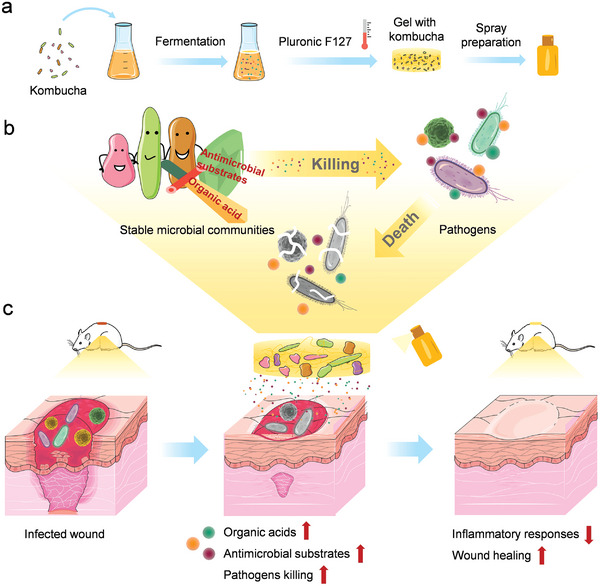
Illustration showing the preparation and anti‐infection treatment of the biotherapeutic gel. a) The preparation of Pluronic F127‐based biotherapeutic gel (gel with kombucha) that contains stable microbial communities (kombucha). b) The anti‐infection mechanism of gel with kombucha against pathogens. c) Application of gel with kombucha in infected wounds.

Here, we tried to explore whether kombucha could be applied to control bacterial infections through social intelligence. First, we regulated the growth rate of microbial communities and investigated their growth stability. Subsequently, we evaluated the antimicrobial efficacy of kombucha against both bacteria and fungi, and further identified the bioactive fractions that might contribute to the antibacterial effect of kombucha. Toward better practical applications, we engineered a Pluronic F127 (polyethylene‐polypropylene glycol) ‐based in situ sprayed biotherapeutic gel (denoted as gel with kombucha) that contained stable microbial communities to eliminate pathogens in infected wounds (**Scheme** **1**). F127 is a highly biocompatible material approved by the United States Food and Drug Administration that has been widely applied in nanomedicine due to its excellent merits including thermosensitive gelatinizing properties, skin adherence, and convenient spray preparations. Thus, gel with kombucha could be sprayed and quickly formed in situ to create a temporary shield for injured tissue, eventually serving as a living biotherapeutic to repel pathogens. We identified the superior anti‐infection efficacy of the biotherapeutic gel system to the commercial broad‐spectrum antibacterial gel (0.1% polyaminopropyl biguanide) on inflammatory responses, angiogenesis, and wound closure in diverse pathogenic bacteria‐derived xenograft infection models, as well as in patient‐derived drug‐resistant bacterial xenograft models.

## 2 Results and Discussion

### 2.1 Characterization of Kombucha

**Figure 1:**
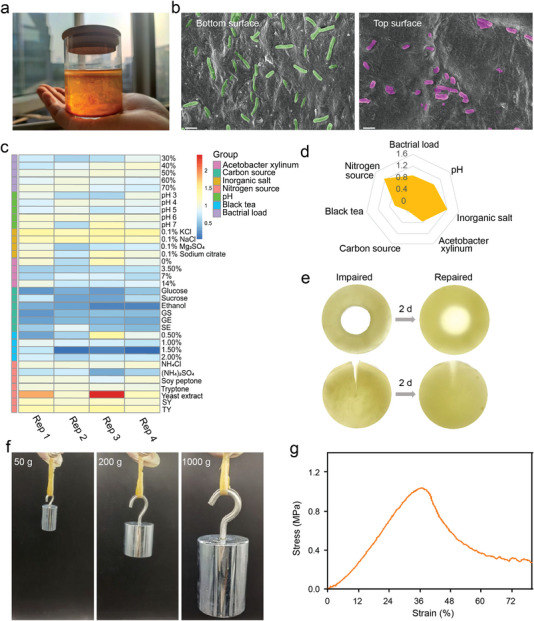
Characterization of kombucha. a) Fermented kombucha tea. A newly formed thinner layer at the surface. b) Scanning electron microscope (SEM) images of the bottom surface (left) and the top surface (right) of the pellicles (scale bar: 2 µm). c) Heat map of nutrition component involved in the growth of the pellicles (GS: glucose and sucrose; GE: glucose and ethanol; SE: sucrose and ethanol; SY: soy peptone and tryptone; TY: tryptone and yeast extract). d) Radar map for the contribution of different nutrition components to the growth of the pellicles. e) Artificial damages to the pellicles were repaired after 2 d. f) Load‐bearing capacity of the pellicles. g) Stress‐stretch curve of the pellicles.

Kombucha is a fermented product by a microbial community commonly referred to as a symbiotic culture of bacteria and yeast. When grown in static cultures, they continuously secreted numerous individual glucan chains, which were then bundled into cellulose fibrils, ultimately “growing” a thick pellicle at the interface between the medium and the air (**Figure** **1**a). Bacterial cellulose and embedded microorganisms were held together tightly by van der Waals forces and hydrogen bonds. The overt difference in microbial shape between the bottom and top of the pellicles indicated that microorganisms in the pellicles might arrange specific ecological niches depending on their oxygen demand (Figure 1b). To investigate the controllability of bacterial growth, we first identified conditions in which symbiotic consortiums could be efficiently co‐cultured. This required screening for growth in different media, including bacterial load, pH, inorganic salts, carbon source, and the black tea proportion. We measured the thickness and time of pellicle formation to determine the growth enhancement and rate of the kombucha, respectively. Notably, evidence showed that the growth enhancement was conferred by the nitrogen source, while the growth rate was mainly governed by the carbon source. Therefore, the nitrogen source and carbon source were supplemented based on the black tea in the following assays (Figure 1c,d; Figures S1, S2, Supporting Information). The movement of bacteria was accompanied by the biosynthesis of cellulose, culminating in the formation of a highly developed fine network of woven structures.^[^ ^24^, ^25^ ^]^ To further explore the self‐repair possibility of the pellicle, we artificially created gaps in the intact pellicle, followed by supplying a nutrient medium to the damaged area. After 2 days, a dense, concrete‐like layer that filled the damaged furrow was observed, thus highlighting that the living composites could confer enhanced durability for repair applications (Figure 1e). In addition, stress‐stretch assays showed that Young's moduli of the pellicle were 3.8 MPa, indicating that the ultrapure nature of bacterial cellulose afforded excellent mechanical properties (Figure 1f,g). During the long‐term co‐culture dynamics, the odor of the fermentation broth and the morphology of the pellicles were consistent. Thus, we assumed that these phenomena were attributed to the existence of stable flora.

### 2.2 Stability of the Symbiotic Consortiums

**Figure 2:**
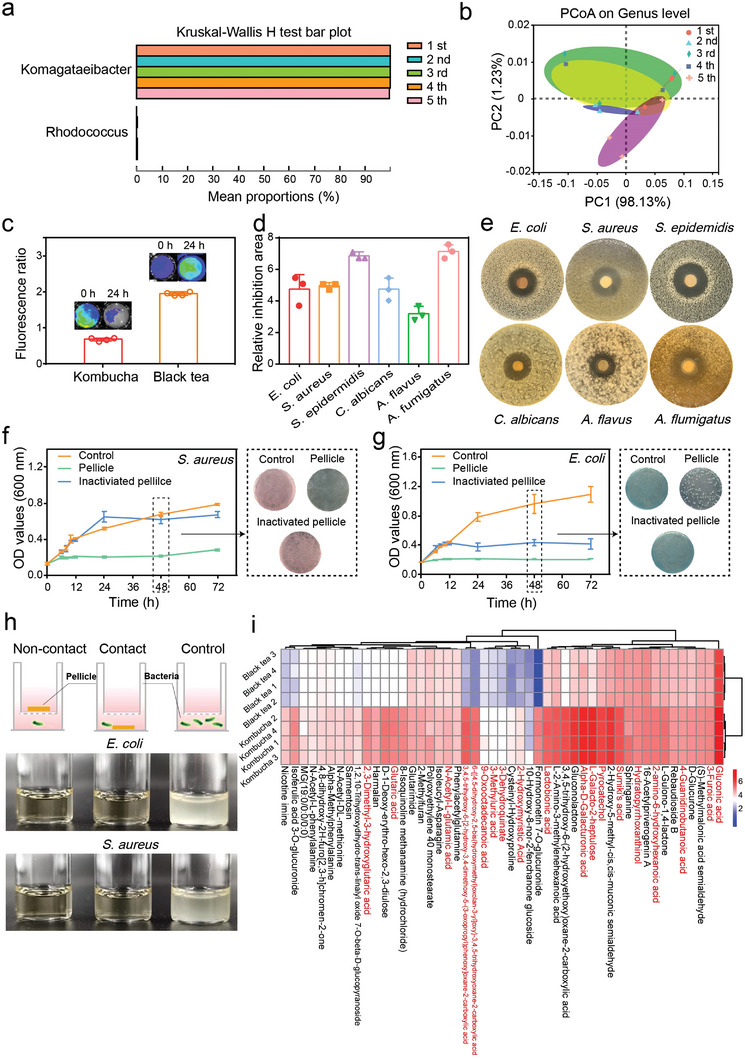
Antagonism ability of the kombucha‐based symbiont against foreign bacteria. a) 16S ribosomal RNA sequencing analysis of passaged pellicles. b) PCoA analysis on genus level of passaged pellicles. c) 24 h fluorescence ratio after mCherry fluorescence expressing *E. coli* co‐culture with kombucha or black tea. d) Quantification of the relative inhibition area (n = 3). e) Zones of inhibition surrounding the kombucha‐infused paper disks against *S. epidermidis*, *E. coli*, *S. aureus*, *C. albicans*, *A. flavus* and *A. fumigatus*. f,g) Growth curves of *S. aureus* and *E. coli* after different treatments indicated. Bacterial colonies were grown on agar plates of *S. aureus* and *E. coli* after coculturing with the pellicle for 48 h. The error bars showed the standard deviation (n = 3). h) Schematic diagram of transwell chamber for simulating non‐contact and contact inhibition between microorganisms. i) The top 50 upregulated compounds of the medium after kombucha fermentation.

We first confirmed the genus‐level resolution analysis of microbiota on serially passaged pellicles. The abundance of *Komagataeibacter* was significantly higher than other strains and there was no significant distinction in community richness (**Figure** **2**a; Figure S3, Supporting Information). The results of PCoA showed that the samples between the groups were very close, and the R^2^ and *P* values were analyzed to be 0.3025 and 0.388 by ANOSIM/Adonis, respectively, indicating that there was no statistical difference in species composition between the groups (Figure 2b; Figure S4, Supporting Information).

### 2.3 Antagonism Ability of Kombucha against Foreign Bacteria

Based on the above results, we confirmed that kombucha was a stable co‐culture system for months in the exposed environment, and further speculated that such an ingenious symbiont owned the ability to repel foreign bacteria and maintain the original homeostasis through microbial social intelligence. Therefore, we further investigated the ability of this symbiont to resist foreign bacteria. Contact sterilization experiments were performed by immersing kombucha (experimental group) or black tea (control group) into mCherry fluorescence‐expressing *Escherichia coli* (*E. coli)* culture solutions to assess the antibacterial property of kombucha. The mCherry fluorescence intensity of bacteria cultured for 24 h was measured. As shown in Figure 2c, the fluorescence intensity of *E. coli* decreased in the experimental group, while increased significantly in the control group, indicating that the growth of *E. coli* was partially inhibited by kombucha (Figure S5, Supporting Information). Respiratory infections are often accompanied by either bacterial or fungal coinfections. Therefore, a range of pathogens including *Staphylococcus epidermidis* (*S. epidermidis*, ATCC 12228), *E. coli* (ATCC 25922), *Staphylococcus aureus* (*S. aureus*, ATCC 6538), *Candida albicans* (*C. albicans*, ATCC 10231), *Aspergillus flavus* (*A. flavus*, ATCC MYA‐3631) and *Aspergillus fumigatus* (*A. fumigatus*, ATCC 96918) were chosen to evaluate the anti‐infective efficacy of the symbiont. Filter paper disks infiltrated with black tea or kombucha were prepared, and an agar disk diffusion test was performed to assess the susceptibility of the aforementioned bacteria or fungi to these materials. Significant inhibition zones of bacteria and fungi were observed around the paper disks infiltrated with kombucha, suggesting the broad antimicrobial spectrum and robust antibacterial effect of kombucha (Figure 2d,e; Figure S6, Supporting Information). The antibacterial properties of black tea and kombucha with different pH values against *E. coli* and *S. aureus* were also assessed. The results revealed that free hydrogen ions had no significant antibacterial properties, and the antibacterial properties of organic acids and antibacterial active substances showed pH dependence (Figure S7, Supporting Information). As two of the most wildly implicated pathogens in wound infection, *S. aureus* (Gram‐positive, ATCC 12228) and *E. coli*, (Gram‐negative, ATCC 25922) were chosen to evaluate the dynamic antibacterial properties of kombucha. Bacterial solutions co‐cultured with the pellicles or inactivated pellicles were established as the experimental groups, while bacterial solutions were used as the control groups. Optical density at 600 nm (OD_600_) of bacteria cultured at different times was measured. As shown in Figure 2F,G, the OD_600_ values of *S. aureus* and *E. coli* in the control groups increased significantly within 24h and reached a peak at 72 h. In the presence of inactivated pellicles, *S. aureus* showed a similar growth trend to the control groups, while *E. coli* exhibited a significant growth inhibition. The difference might be ascribed to the selective mechanisms of bacterial cellulose on bacteria. In the pellicle groups, the OD_600_ values of *S. aureus* and *E. coli* almost did not increase within 72 h, indicating that the bacterial growth was completely inhibited. The turbidity degree of bacterial culture solutions was related to the number of bacteria. It was observed that the culture solutions or solutions with inactivated pellicles were cloudy while those with pellicles were clear after 48 h, which was consistent with the results from bacterial growth curves (Figure S8, Supporting Information). Meanwhile, 100 µL of the bacterial suspension was uniformly spread on an available chromogenic agar plate. After being incubated on an agar plate at 37 °C for another 24 h, the bacterial colony‐forming units (CFUs) were counted. The agar plates of the pellicle groups hardly showed pink colonies, indicating that *S. aureus* was almost completely killed. Similar situations also appeared in *E. coli*. In addition, our experimental results showed that there were certain differences in the antibacterial properties of kombucha fermented from different tea leaves (Figure S9, Supporting Information). It was worth noting that kombucha exhibited a significant inhibitory effect on methicillin‐resistant *S. aureus* and was expected to be an alternative for the treatment of drug‐resistant bacterial infections (Figure S10, Supporting Information). Taken together, these results convincingly suggested that kombucha exhibited a highly effective and long‐lasting antibacterial effect.

### 2.4 Antimicrobial Mechanism of Kombucha

All major bacterial phyla have now been shown to possess antagonistic pathways, including both contact‐dependent and contact‐independent mechanisms in many cases.^[^ ^26^, ^27^ ^]^ To evaluate the bacterial antagonism mechanism between kombucha and foreign bacteria, *E. coli* and *S. aureus* was co‐cultured with the pellicles embedded with kombucha by contact or non‐contact methods, respectively (Figure 2h). The results showed that the liquid remained clear in both methods, demonstrating that there was non‐contact inhibition of kombucha against *E. coli*. To further explore which active substances mediated the interbacterial antagonistic interactions, we then performed non‐targeted metabolomics to compare the metabolic profiles of kombucha fermentation broth and black tea, while evident changes in the global metabolite profile were observed (Figure S11, Supporting Information). Enrichment analysis by KEGG pathways revealed that differential metabolites were mainly involved in the metabolism of amino acids, carbohydrates, lipids, and nucleotide metabolisms (Figure S12, Supporting Information). Moreover, the top 50 upregulated compounds were selected and displayed in the heatmap based on their relative abundance (Figure 2i). As a result, the composition of organic acids in the fermentation broth increased greatly, contributing to a low pH environment unfavorable for most acid‐intolerant bacteria. More importantly, hydratopyrrhoxanthinol,^[^ ^28^, ^29^ ^]^ *L*‐galacto‐2‐heptulose,^[^ ^30^ ^]^ pyrocatechol,^[^ ^31^, ^32^ ^]^ lactobionic acid^[^ ^33^, ^34^ ^]^ and 3‐dehydroquinate^[^ ^35^, ^36^ ^]^ secreted by kombucha were identified to possess potential antibacterial properties. Collectively, these data illustrated that metabolites produced in the kombucha fermentation broth could form an antimicrobial environment to maintain a stable symbiotic consortium. Despite these advances, the antimicrobial effectiveness of kombucha was attributed to multitudinous antimicrobial substances and organic acids, making it challenging to decipher the accurate antimicrobial mechanism of kombucha. How to identify the components of kombucha more completely and accurately screened the dominant active ingredients with high throughput could be further explored in the future.

### 2.5 Performance Determination of the Biotherapeutic Gel

**Figure 3:**
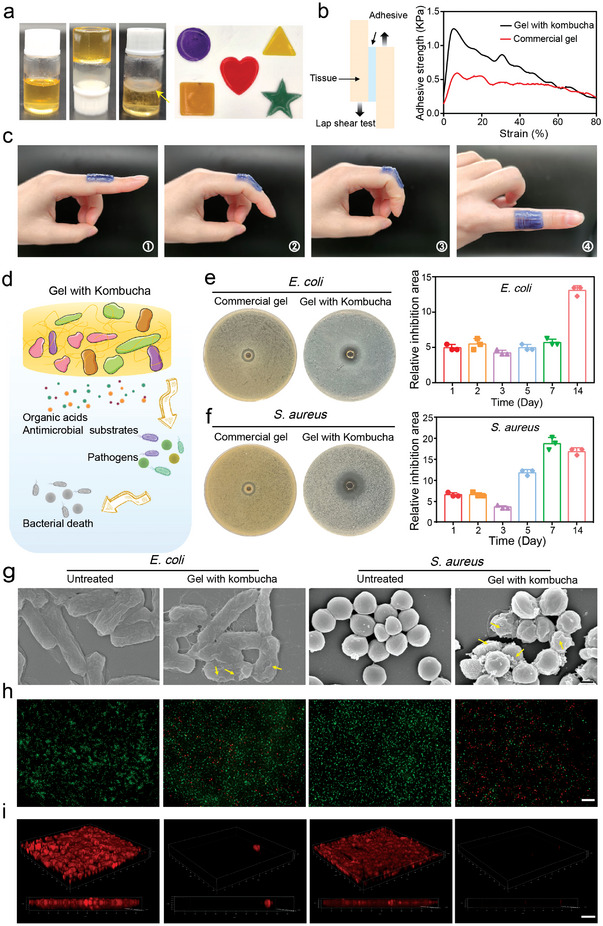
Performance determination of the biotherapeutic gel. a) Reversible transformation of gel with kombucha (22% w/v). b) Adhesive strength versus strain curves for lap‐shear tests of tissues adhered by the different gels. c) Digital photos of gel with kombucha adhered to the frequently moving joints of the body. d) Purposed antimicrobial mechanism of gel with kombucha against pathogens. e,f) Zones of inhibition surrounding gel with kombucha or commercial gel against *E. coli* and *S. aureus*. Quantification of the relative inhibition area on day 7 (n = 3). g) The morphology transition of pathogens after the treatment of gel with kombucha (scale bar: 400 nm). h) Bacteria staining with MycoLight Green (green) and PI (red) after gel with kombucha treating for 12 h (scale bar: 20 µm). i) CLSM 3D images of *E. coli* and *S. aureus* biofilms in the presence of gel with kombucha (scale bar: 75 µm).

We hypothesized that a stable microbial community capable of continuously producing antibacterial molecules to compete for limited nutrition or space for survival might be an alternative treatment for superficial bacterial infections. Toward better practical applications, we designed a biotherapeutic gel (denoted as gel with kombucha) containing the kombucha and a thermo‐responsive polymer F127, which was a liquid at room temperature and quickly solidified on the skin after spraying due to its lower critical solution temperature close to, body temperature.^[^ ^37^, ^38^, ^39^ ^]^ The newborn pellicle in the gel indicated that kombucha could grow in the polymer F127, showing the good biocompatibility of F127. Due to the good fluidity and rapid gelation properties, the biotherapeutic gel could be flexibly formed into any shape (**Figure** **3**a). To evaluate the potential utilization of kombucha gel as a medical adhesive, lap shear testing was performed using porcine skin (Figure 3b). The elastic modulus of the gel with kombucha and the commercial gel was measured to be 37.1 and 19.6 KPa, respectively, demonstrating that the adhesin strength of the gel with kombucha was higher than that of the commercial gel. To further verify the repeatability and stability, the gel with kombucha was fixed on the dynamic skin surfaces and underwent stretching. The position of the hydrogel was found to fix without any retraction or rupture during the testing process (Figure 3c). These properties enable the gel to undertake versatile geometries to suit various end effectors such as joints, and to form fluid‐tight seals with curved and irregular tissue surfaces. The antibacterial effect of the “live” gel was further evaluated via agar disk diffusion assay. As for *E. coli*, the relative inhibition area remained almost unchanged after 7 days and increased significantly after 14 days, indicating that the active antibacterial substances secreted by kombucha were accumulated to enhance the antibacterial effect over time. In terms of *S. aureus*, the relative inhibition area continued to increase within 7 days and remained at a stable level, implying the enduring antimicrobial effect of kombucha (Figure 3d–f). The morphologies of treated bacteria were obtained by SEM. In the untreated group, integrated and smooth membranes were shown. In contrast, *E. coli* treated with gel with kombucha were seriously impaired with irregularly shaped cracks (indicated by orange arrows) in the cell walls and exhibited a crumpled morphology. The morphological destruction was more pronounced in *S. aureus* as many bacterial fragments could be observed (Figure 3g). It indicated that kombucha could destroy the structure of bacterial peptidoglycan or inhibit its synthesis, both of which damaged the cell wall and deformed or killed the bacteria. Meanwhile, live/dead cell viability assays were further used to establish the killing effect of gel with kombucha against *E. coli* and *S. aureus*. More bacteria were stained as red fluorescence in the gel with kombucha group compared with the untreated group, indicating the efficient killing performance of gel with kombucha against typical pathogens (Figure 3h; Figure S13, Supporting Information). Notably, biofilm formation plays an important role in the development of infection and is a major reason for drug resistance. The inhibitory effects of gel with kombucha on the biofilm formation of *E. coli* and *S. aureus* were further investigated. To visualize the biofilm inhibitory effect of gel with kombucha, dead cells in the biofilm samples were stained with ethidium bromide. Under confocal laser scanning microscope (CLSM), the bacterial biofilm in the untreated group was densely colonized, forming a hierarchical and 3D structure, as evident in Figure 3i. In contrast, significantly reduced bacterial cells and scattered biofilms were observed after the treatment of gel with kombucha, indicating excellent inhibition efficiency against biofilm formation. To quantify the inhibition of biofilm formation, bacterial cell metabolic activity was detected by crystalline violet staining. The biofilm volume of the untreated group was defined as 100%. The inhibition rate of biofilm formation by gel with kombucha was 93% for *E. coli* and 82% for *S. aureus*, respectively (Figure S14, Supporting Information). Additionally, the biofilm elimination effect was examined. The complete and dense biofilms formed by *E. coli* and *S. aureus* were treated with gel with kombucha (Figure S15, Supporting Information), only a small amount of bacterial residue remained in the biofilms and the biofilm elimination rate was as high as 59% and 63% for *E. coli* and *S. aureus*, respectively (Figure S16, Supporting Information).

### 2.6 Biosafety Assessment

**Figure 4:**
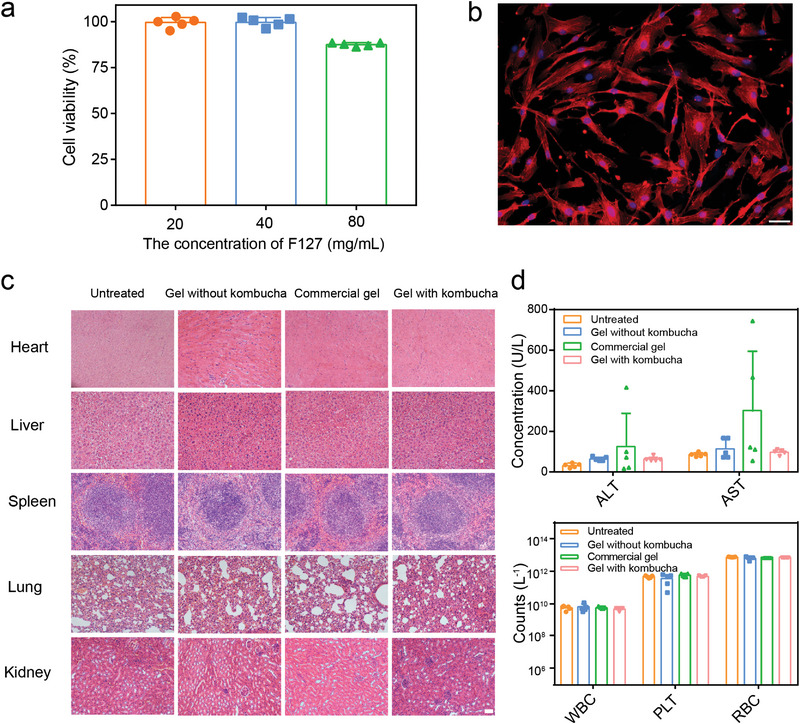
Biosafety assessment. a) Relative cell viability of the HUVEC cells after treatment with different concentrations of F127 (n = 5). b) Phalloidin staining of the actin cytoskeleton architecture in HUVEC after treating with gel with kombucha for 12 h (scale bar: 100 µm). c) H&E staining images of heart, liver, spleen, lung, and kidney sections from mice (scale bar: 100 µm). d) Blood routine examination and blood biochemistry about inflammation, liver, and kidney function indicators in mice treated with different groups (n = 5).

Biosafety is important as well for a potential antibacterial candidate, especially for bacteria‐based biotherapeutics applied in vivo. The biocompatibility of gel with kombucha was then tested in the human umbilical vein endothelial cells (HUVEC) via MTT assay and phalloidin staining (**Figure** **4**a,b). MTT assay substantiated the negligible toxicity of F127 at concentrations below 40 mg mL^−1^. Intact cellular actin cytoskeleton architecture was well reserved after cells were exposed to gel with kombucha as observed from the Phalloidin staining images. In addition, we examined the possible tissue toxicities when applicating gel with kombucha to infectious wounds. Hematoxylin and eosin (H&E) staining of organs (heart, liver, spleen, lung, and kidney) showed no obvious physiological abnormity and detectable tissue damage. Blood routine examination (WBC, PLT, and RBC) and blood biochemistry about inflammation, liver, and kidney functional indicators (ALT, AST, CRE lower than 20 µmol L^−1^ and TBIL lower than 5 µmol L^−1^) revealed that gel with kombucha did not lead to any significant alterations (Figure 4c,d). ALT and AST indexes of some mice in the commercial gel group were beyond the normal range, suggesting that liver injury occurred in the experimental mice, which might be caused by bacterial infection during modeling. These results indicated that this biotherapeutic gel possessed good biocompatibility within the therapeutical dose, which could be applied in the treatment of bacterial infectious wounds.

### 2.7 Healing in a Full‐Thickness Cutaneous Wound Model

**Figure 5:**
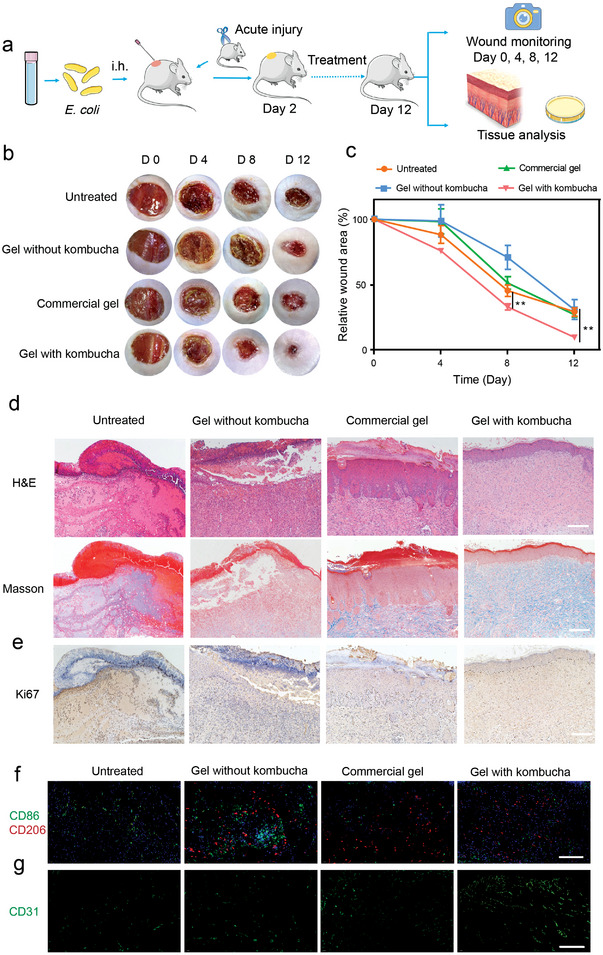
Evaluation of the healing in a full‐thickness cutaneous wound model. a) Schematic illustration of steps for a full‐thickness cutaneous wound model with *E. coli* infection. b) Representative images of wound tissues after different treatments on days 0, 4, 8, and 12. c) Relative wound area analysis over time in mice after various treatments (n = 4). d) H&E and Masson staining of the wound areas in the different groups on day 9. e) Immunohistochemical images of Ki67 in wounds (scale bar: 200 µm). f) Immunofluorescence images of M1 (CD86) and M2 (CD206) macrophages markers on day 3 (scale bar: 200 µm). g) Immunofluorescence images of CD31 angiogenesis markers on day 9 (scale bar: 200 µm). **P < 0.01; The significance between every two groups was calculated using one‐way ANOVA (c). Mean values ±s.d. are presented.

Inspired by the shape flexibility and ease of adhesion as well as the remarkable in vitro antibacterial efficacy of the biotherapeutic gel, we then estimated its potency in vivo and potential application as wound dressings. A full‐thickness cutaneous wound model with *E. coli* infection was established first to evaluate the performance of the biotherapeutic gel in promoting wound healing (**Figure** **5**a). Infected mice were divided into four groups: untreated, gel without kombucha, commercial gel, and gel with kombucha. Digital photographs of wounds showed that mice treated with gel with kombucha exhibited a much faster wound healing rate compared to the other groups (Figure 5b,c). Significant differences were observed between the gel with kombucha and the commercial gel groups. These results implied the excellent behavior of kombucha in promoting wound healing, which was attributed to the excellent antibacterial ability of the biotherapeutic gel by secreting bioactive metabolites. The wound samples extracted from the treated mice were then processed for further pathologic analyses. H&E staining showed that wounds in the untreated and gel without kombucha groups presented ulcer formation on the wound epidermis with inflammatory cell infiltration, and no squamous epithelial coating was observed, indicating that the wounds were still in the inflammatory stage. In commercial gel‐treated wounds, squamous epithelial cell coating was visible but hyperkeratotic, which was associated with acute and chronic inflammatory cell infiltration, implying excessive wound repair. Despite a small amount of inflammatory cells infiltration in gel with kombucha‐treated wounds, the wound epidermis presented a multilayered epithelium structure that closely resembled the healthy epidermis of the intact skin, demonstrating good repair (Figure 5d). Masson and immunohistochemical staining for the proliferative marker ki67 indicated that wounds treated with gel with kombucha manifested much higher levels of collagen deposition and skin cell proliferation than those treated with gel without kombucha or commercial gel. The high expression of Ki67 in the wounds of the untreated group might be due to inflammation (Figure 5e). Macrophages are known to play important roles from the initiation of inflammation to wound healing. Two phenotypes of macrophages, namely pro‐inflammatory M1 macrophages and anti‐inflammatory M2 macrophages have been identified. CD86 and CD206 have been widely used as markers for M1 and M2 macrophages, respectively.^[^ ^40^, ^41^, ^42^, ^43^ ^]^ We next examined the inflammatory responses in the wound areas. As shown in Figure 5f, untreated wounds and wounds treated with gel without kombucha were populated by CD86, while wounds treated with commercial gel and gel with kombucha were populated by CD206. These results suggested that gel with kombucha could polarize macrophages from M1 to M2 phenotype, and a more subdued inflammatory status would facilitate wound healing. In addition, CD31 immunostaining further supported the role of gel with kombucha in promoting angiogenesis during the healing process (Figure 5g; Figure S17, Supporting Information). These results revealed that the biotherapeutic gel could effectively control inflammation and promote cell proliferation as well as angiogenesis, thus eventually accelerating wound healing.

### 2.8 Healing in a Burn Wound Model

**Figure 6:**
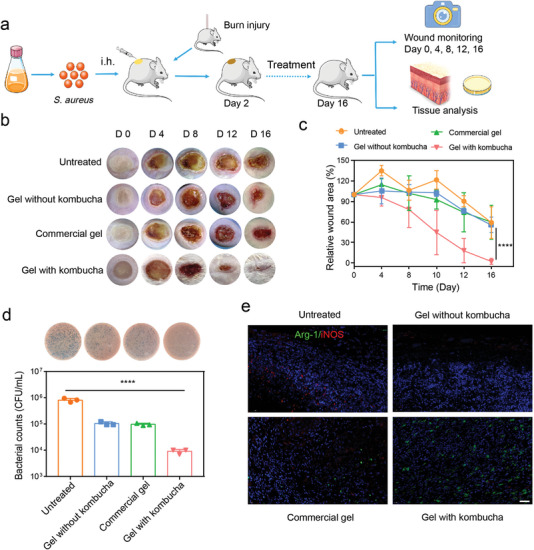
Evaluation of the healing in a burn wound model. a) Schematic illustration of steps for a burn wound model with *S. aureus* infection. b) Representative images of wound tissues by different treatments on days 0, 4, 8, 12, and 16. c) Relative wound area analysis over time in mice after various treatments (n = 4). d) Bacterial burden around the infected wound site on day 6 was measured using the chromogenic agar plate, and CFU quantitative analysis (n = 3). e) Immunofluorescence images of Arg‐1 (green) and iNOS (red) on day 6 (scale bar: 40 µm). ****P < 0.0001; The significance between every two groups was calculated using one‐way ANOVA (c,d). Mean values ±s.d. are presented.

To investigate whether the in situ sprayed biotherapeutic gel can provide therapeutic benefits to burn wounds, two full‐thickness burn wounds were created on the back of each mouse followed by subcutaneous injection of *S. aureus* (**Figure** **6**a).^[^ ^44^, ^45^ ^]^ The wounds of mice in the different groups were monitored and photographed with a digital camera. Yellow pus was observed at the wound site of almost all mice on day 4, and the injury was bright red, indicating that the burn site was undergoing inflammation. However, there was almost no yellow pus in the gel with kombucha groups, while continuous inflammation was still observed in the other groups. During the entire treatment period, the wounds of mice treated with gel with kombucha exhibited accelerated wound closure, indicating that the infection caused by *S. aureus* was well controlled. Meanwhile, the wound areas of mice in the untreated and gel without kombucha groups showed repeated decrease–increase, indicating recurrent infections. The wound healing rate of the commercial gel group was significantly slower than that of the gel with the kombucha group (Figure 6b,c). We next explored the impact of gel with kombucha on pathogen colonization in wound areas. The wound samples were homogenized, and the extracts were subjected to bacterial colony counting on agar plates. As shown in Figure 6d, a large number of bacterial colonies were observed in the untreated group. Both groups of gel without kombucha and commercial gel were found reduced bacterial colonization to a certain extent, and the number of bacteria in wounds treated with gel with kombucha was the lowest, demonstrating the superior inhibitory effect of gel with kombucha on pathogen colonization. Furthermore, inducible nitric oxide synthase (iNOS), and arginase‐1 (Arg‐1), have been identified as markers for M1 and M2 macrophages, respectively.^[^ ^46^, ^47^ ^]^ The iNOS/Arg‐1 expression ratio in the gel with kombucha group significantly decreased compared with that in the other groups, suggesting the fact that gel with kombucha could induce macrophage polarization from M1 to M2 phenotype during the process of wound healing (Figure 6e; Figure S18, Supporting Information). The shift in macrophage polarity is beneficial for the transition from inflammation to a healthy state.

### 2.9 Healing in Patient‐Derived Xenograft Infections Models

**Figure 7:**
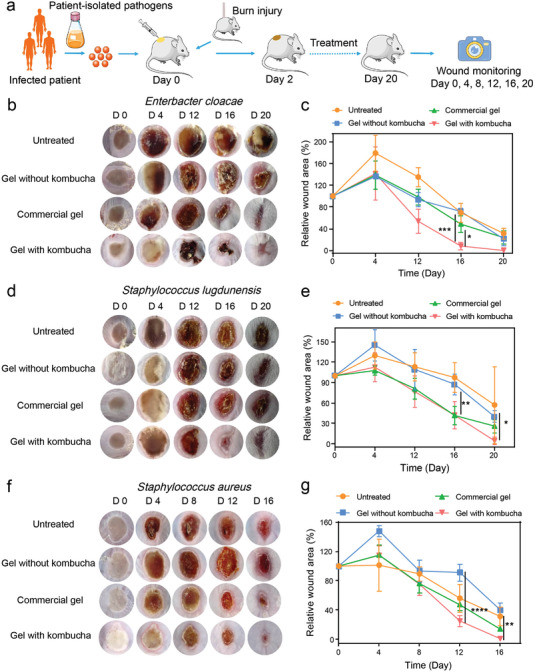
Evaluation of the healing in patient‐derived xenograft infections models. a) Schematic illustration of the experimental process used to evaluate anti‐infection therapy in vivo using a patient‐derived xenograft infectious model. b–g) Representative images of wound tissues infected by different patient‐isolated pathogens after various treatments indicated (from top to bottom, *E. cloacae, S. lugdunensis, S. aureus*. n = 4). *P < 0.05, **P < 0.01, ***P < 0.001, and ****P < 0.0001; The significance between every two groups was calculated using one‐way ANOVA (c,e,g). Mean values ±s.d. are presented.

To further validate the potential clinical utility of the constructed biotherapeutic gels in the treatment of drug‐resistant bacterial infections, we established a patient‐derived xenograft infectious model (**Figure** **7**a). Initially, multidrug‐resistant pathogenic bacteria from patients with recurrent infected wounds were collected, tested, and subsequently used for mouse burn wound infections (Table S1, Supporting Information). Specifically, *Enterobacter cloacae* (*E. cloacae*), *Staphylococcus lugdunensis* (*S. lugdunensis*), *S. aureus* were used as the pathogens for verification. After the transplantation of these three pathogenic bacteria into the burn wounds separately, four groups the same as the above models were administered for treatments. As shown in Figure 7b–g, pus and redness were shown in wounds after transplantation with patient‐derived pathogens. Although the infection degree with pathogens from different patients was slightly diverse, the use of gel with kombucha still exhibited apparent therapeutic efficacy, demonstrating the ability of gel with kombucha to kill drug‐resistant bacteria and promote wound healing. The severity of various resistant pathogen‐induced infections and the pathogen sensitivity to the active antibacterial substances secreted by kombucha were the dominant causes responsible for the therapeutic effect. Notably, gel with kombucha exhibited comparable or even superior therapeutic efficacy to the commercial gel against drug‐resistant bacterial infections, indicating the considerable clinical translation potential of such an in situ sprayed biotherapeutic gel containing stable microbial communities. It should be mentioned that our work confirmed the broad‐spectrum antimicrobial properties of kombucha in vitro, including both bacteria and fungi. Unfortunately, no fungal samples were collected from the infected wound of the patients during the sample collection period, so the therapeutic effect of kombucha in drug‐resistant fungi infection did not be verified in vivo.

## 3 Conclusion

In this study, we have demonstrated the potential for utilization of an in situ sprayed biotherapeutic gel containing a stable symbiotic consortium for infectious wound healing. The stable microbial communities obtained survival advantage by secreting active antimicrobial substances, thus possessing remarkable antagonistic abilities against a wide range of pathogens both in vitro and in vivo. The in situ sprayed biotherapeutic gel possessed excellent shape flexibility and surface adherence as well as high biocompatibility, which could be expediently processed to serve as long‐acting antibacterial dressings for wound management. This biotherapeutic gel was then demonstrated to exhibit excellent therapeutic benefits in multidrug‐resistant pathogen‐induced wound healing via persistent anti‐infection. Symbiotic consortiums capable of self‐stabilization and antagonism of foreign bacteria activity could effectively treat infections while avoiding systemic inflammation caused by bacteria, providing an alternative to antibiotics for treating infection. This work may not only make a good attempt for the application of bacterial social dynamics to interfere with infectious diseases, but open the door for deeper exploration and understanding of the application of bacteria‐based biotherapeutics in biomedicine.

## 4 Experimental Section

### Preparation of Kombucha

The original kombucha was derived from a local village in the Fujian province of China and maintained at 25 °C in a sterilized medium containing tea and sucrose. At the end of the designated periods, the fermented samples were collected for further characterization.

### Bacterial Strains and Culture

*S. epidermidis* (ATCC 12228), *E. coli* (ATCC 25922), *S. aureus* (ATCC 6538), *C. albicans* (ATCC 10231), *A. flavus* (ATCC MYA‐3631) and *A. fumigatus* (ATCC 96 918) were obtained from Guangdong Microbial Culture Collection Center. Bacteria were cultured under recommended conditions before experiments.

### Patient Samples

Infectious patient samples were obtained from the Zhongnan Hospital in Wuhan, China. The study (2022044K) was approved by the ethical review board at Zhongnan Hospital, and signed informed consent was obtained from patient donors.

### Characterization of Microbial Communities

A scanning electron microscope system (Carl Zeiss sigma 500, Germany) was employed to observe the microstructure of the samples. Uniaxial tensile tests of these gels were carried out by using a commercial tensile tester (Instron 5966). An abundance of bacteria was analyzed by 16S ribosomal RNA sequencing. Identification of bioactive substances in samples was performed by untargeted metabolomics analysis.

### Agar Disk Diffusion Assay

Antimicrobial susceptibility tests were conducted by the agar disk diffusion assay. Briefly, the 6‐mm diameter filter paper disks were pre‐soaked with kombucha or black tea. The bacterial suspensions were evenly swabbed onto agar plates and the pretreated paper disks were placed onto the plates. After incubation at 37 °C for 24 h, the plates were photographed and the diameters of inhibition zones were measured. As for the antibacterial agar disk diffusion assay of the gel, a hole punch was used to create a 6 mm round hole for placing the sample.

### Bacterial Growth Curves

*S. aureus* and *E. coli* were used as model strains to investigate the antibacterial activity of microbial communities. The original bacterium fluid was inoculated in an LB growth medium for 72 h at 37 °C with constant shaking. In this assay, inactivated pellicles were used as the control groups. Each assay was performed in triplicate. During the incubation, the optical density values at 600 nm of the bacterial suspensions were monitored at different time points. Besides, 100 µL of the bacterial suspension was uniformly spread on an available chromogenic agar plate. After being incubated on agar plates at 37 °C for another 24 h, the CFUs were counted. Meanwhile, the turbidity change of bacterial suspension was also photographed.

### Transwell Migration Assay

To identify the antibacterial mechanism, pellicles were placed into the upper chamber or the lower chamber; the lower chamber was added with the bacterial suspensions. After 24 h of incubation, the turbidity change of bacterial suspension was photographed.

### Gel Formation and Gelation Time Determination

Pluronic F127 was dissolved in ultra‐pure water to give a series of final concentrations of 16%, 18%, 20%, 22%, and 24% (w/v). The solutions were all placed in a 37 °C environment. Gelation time was assessed visually by observing the color change and loss of clarity (n = 4). A similar procedure was followed for the 22% (w/v) Pluronic solution containing kombucha in a nutrient medium.

### Biofilm Inhibition

Biofilm inhibition assays were performed as described previously.^[^ ^48^ ^]^ Bacteria were incubated in LB medium at 37 °C overnight and then diluted with LB medium to OD_600_ = 0.05. Subsequently, the bacterial diluent was further combined with an equal volume of gel with kombucha to obtain the final seed solution. An equal volume of untreated bacteria was used as a control. Then, 150 µL of seed solution was added to the wells of 96 plates and co‐cultured statically at 37 °C for 24 h. After the removal of the medium, the formed biofilms were rinsed with PBS. After being completely dried, the biofilms were fixed with anhydrous methanol and then stained with crystal violet solution (0.5%, w/v). Subsequently, acetic acid (33%, v/v) was added to each well and shaken for 15 min to release the dye. The absorbance of each well was measured at 590 nm using a microplate reader to quantify the biofilm. The control was set to 100% of biofilm formation and the inhibition rate of biofilm formation (%) was calculated. the biofilm elimination assays were performed by adding 150 µL of a mixture of gel with kombucha and LB medium after the biofilms were grown in 96‐well plates and incubated for 24 h. The biofilms were quantified using the same method as above.

### Acute Wound‐Healing Mouse Model

Balb/C mice (male, 6–8 weeks) were obtained from Huazhong Agricultural University. All animal experimental procedures were performed in accordance with the Regulations for the Administration of Affairs Concerning Experimental Animals approved by the State Council of the People's Republic of China. A full‐thickness wound was made on the center of the dorsum of Balb/C mice to remove the epidermis and superficial parts of the dermis by using a 10 mm biopsy punch. A 100 µL aliquot of *E. coli* (2 × 10^9^ CFU) in LB medium was smeared on the wound. After 24 h, an abscess with oozing blood formed at the wound, indicating the successful building of the bacterial infection model. The animals were randomly divided into four groups: untreated, commercial gel, gel without kombucha, and gel with kombucha. For therapy, the three groups of mice were treated with commercial gel, gel without kombucha, and gel with kombucha twice a day, respectively. Wound images at the indicated time points were captured. The wound diameters were measured using a caliper rule, and the time for complete wound closure was recorded. At the end of treatment, the blood was taken from mouse hearts for biochemical examinations. The wound tissues were harvested for histological, immunohistochemical, and immunofluorescent analyses.

### Burn Wound‐Healing Mouse Model

A metal rod (10 mm in diameter) was heated to 100 °C by submersion in boiling water. After shaving the mice, the rod was immediately positioned vertically for 20 s on the upper skin of the mice without additional pressure. Then animals received subcutaneous injections of pathogens (100 µL, 2 × 10^8^ CFU) or patient‐derived pathogens (100 µL, 10^7^ CFU). After wounding, the mice received the aforementioned treatments and the wounds were not dressed. On day 6, the homogenates of burn wounds were prepared with a tissue homogenizer and then diluted 1000 times using sterile PBS. The diluted homogenates were spread onto agar plates and incubated at 37 °C. Twenty‐four hours later, the CFU numbers were counted. The wound tissues were harvested for histological, immunohistochemical, and immunofluorescent analyses.

### Immunohistochemical and Immunofluorescent Staining

After being fixed using 4% paraformaldehyde, the paraffin‐embedded tissues were prepared. H&E and Masson staining were used to test re‐epithelization and collagen deposition. Immunohistochemical staining for Ki67 was used to establish cell proliferation. Macrophages were detected by immunofluorescent staining for CD86/CD206 and iNOS/Arg‐1. Antibodies were purchased from Servicebio.

### Statistical Analysis

A Student's *t*‐test (unpaired, two‐tailed) was used for two‐group comparison and one‐ or two‐way analysis of variance (ANOVA) for multiple comparisons. Data were presented as means ±SD. *P* < 0.05 was regarded as statistically significant.

## Conflict of Interest

The authors declare no conflict of interest.

## Supporting information

Supporting Information

## Data Availability

The data that support the findings of this study are available from the corresponding author upon reasonable request.
