# Supplementary material for: In Situ Sprayed Biotherapeutic Gel Containing Stable Microbial Communities for Efficient Anti‐Infection Treatment
Source: Adv Sci (Weinh). 2022 Dec 7;10(4):2205480. doi: 10.1002/advs.202205480 (PMC9896078; doi:10.1002/advs.202205480)
Supplement: Supplementary file 1 — Supporting Information [file ADVS-10-2205480-s001.pdf]

ADVANCED SCIENCE
Open Access

Supporting Information

for *Adv. Sci.*, DOI 10.1002/advs.202205480

In Situ Sprayed Biotherapeutic Gel Containing Stable Microbial Communities for Efficient Anti-Infection Treatment

*Jian-Hua Yan, Di-Wei Zheng, Hui-Yun Gu, Yun-Jian Yu, Jin-Yue Zeng, Qi-Wen Chen, Ai-Xi Yu and Xian-Zheng Zhang**

Supporting information for

In Situ Sprayed Biotherapeutic Gel Containing Stable Microbial Communities for Efficient Anti-Infection Treatment

*Jian-Hua Yan, Di-Wei Zheng, Hui-Yun Gu, Yun-Jian Yu, Jin-Yue Zeng, Qi-Wen Chen, Ai-Xi Yu, and Xian-Zheng Zhang**

Table S1. The data of patient-derived bacterial drug culture and identification

Enterobacter cloacae		*Staphylococcus lugdunensis*		*Staphylococcus aureus*	
Antibiotic	Sensitivity	Antibiotic	Sensitivity	Antibiotic	Sensitivity
Cefazolin	Resistant	Clindamycin	Resistant	Clindamycin	Resistant
Levofloxacin	Sensitive	Ciprofloxacin	Sensitive	Ciprofloxacin	Sensitive
Amikacin	Sensitive	Erythrocin	Resistant	Erythrocin	Resistant
Gentamicin	Sensitive	Gentamicin	Sensitive	Gentamicin	Sensitive
Ciprofloxacin	Sensitive	Levofloxacin	Sensitive	Levofloxacin	Sensitive
Cefoxitin	Resistant	Linezolid	Sensitive	Linezolid	Sensitive
Cefoxitin	Sensitive	Moxifloxacin	Sensitive	Moxifloxacin	Sensitive
Cefoxitin	Sensitive	Oxacillin	Sensitive	Oxacillin	Sensitive
Aztreonam	Sensitive	Penicillin	Resistant	Penicillin	Resistant
Amoxicillin	Resistant	Dalfopristin	Sensitive	Dalfopristin	Sensitive
Imipenem	Sensitive	Rifampicin	Sensitive	Rifampicin	Sensitive
Selectrin	Sensitive	Selectrin	Sensitive	Selectrin	Sensitive
Piperacillin	Sensitive	Tetracycline	Sensitive	Tetracycline	Sensitive
Ertapenem	Sensitive	Tigecycline	Sensitive	Tigecycline	Sensitive
Tigecycline	Sensitive				
Tobramycin	Sensitive				

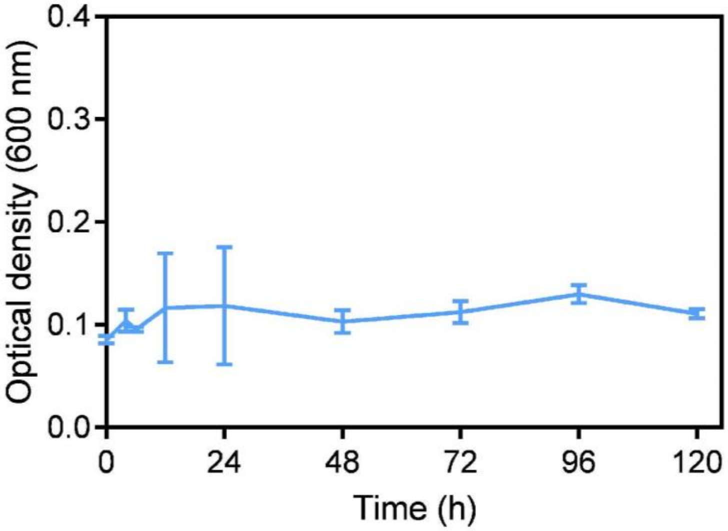

Figure S1. Growth curve of kombucha.

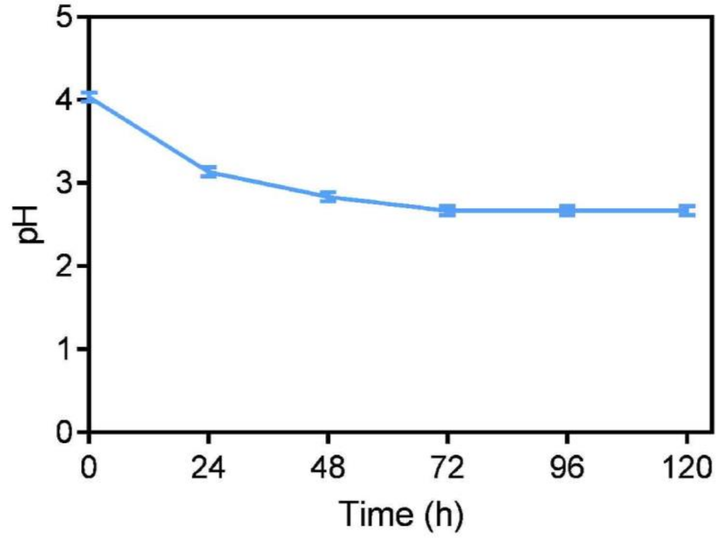

Figure S2. pH curve of kombucha.

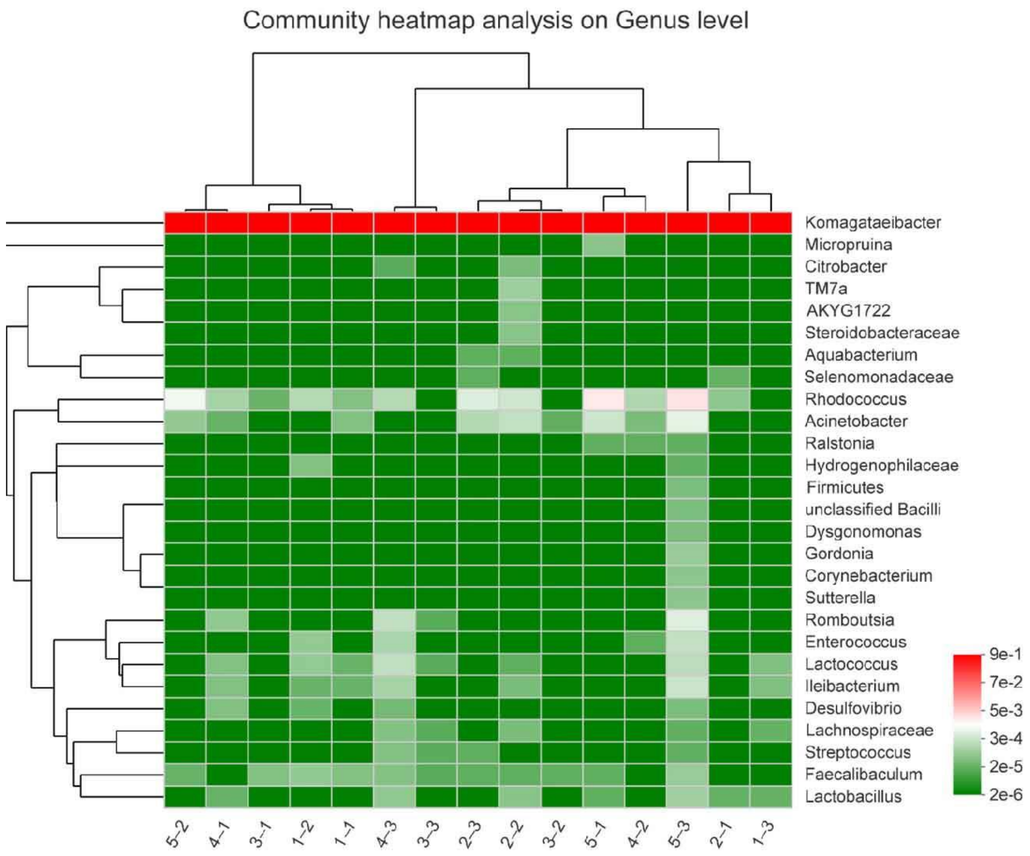

Figure S3. The genus-level relative microbial abundance of serially passaged pellicles.

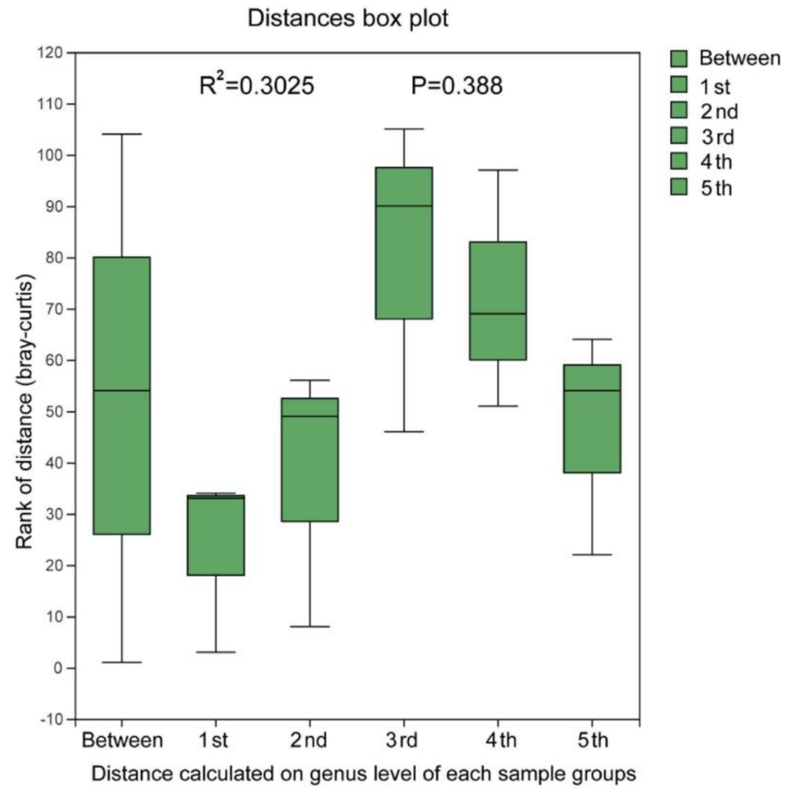

Figure S4. ANOSIM/Adonis analysis of bacterial flora in serially passaged pellicles.

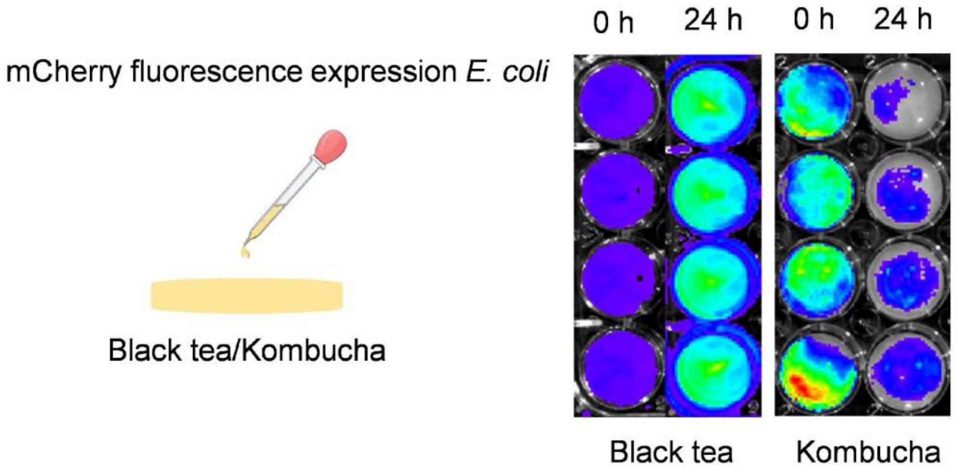

Figure S5. Fluorescence intensity changes of mCherry fluorescence expressing *E. coli* after being cocultured with kombucha or black tea for 24 h.

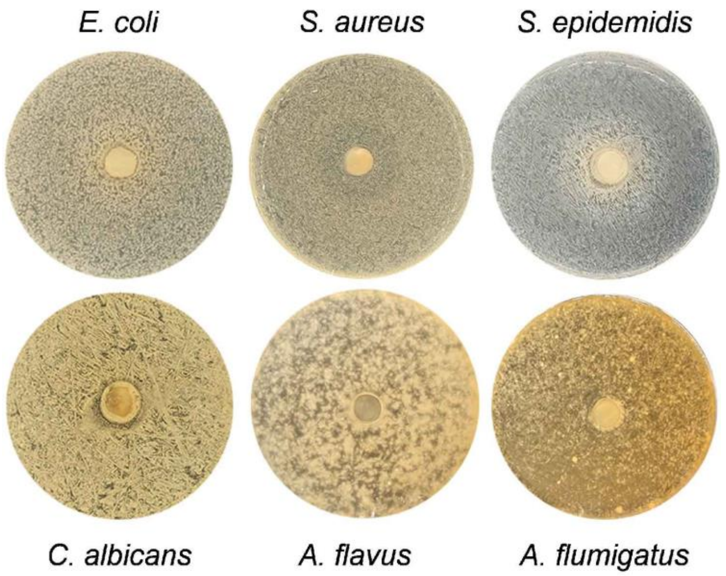

Figure S6. Inhibition zones surrounding the black tea-infused paper disks against *S. epidermidis*, *E. coli*, *S. aureus*, *C. albicans*, *A. flavus* and *A. flumigatus*.

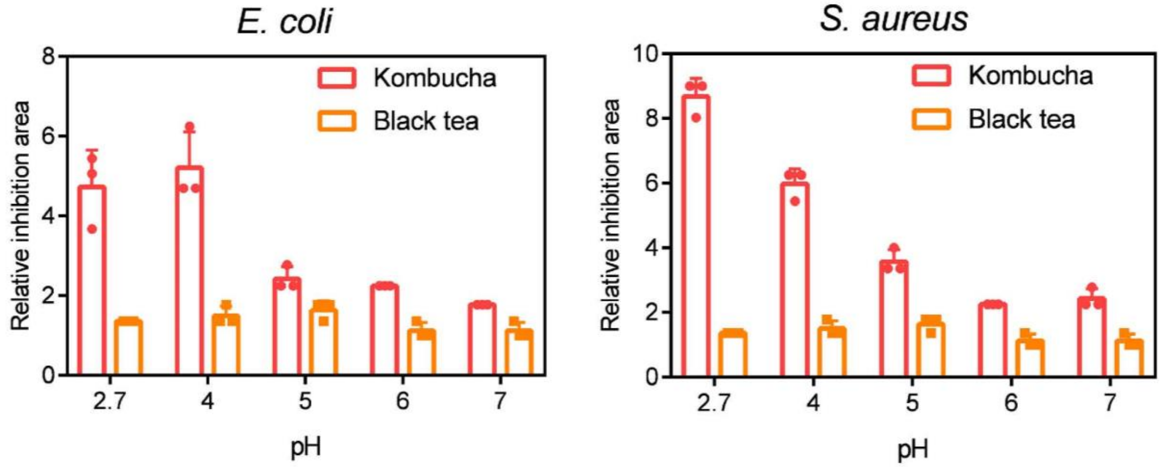

Figure S7. Inhibition zones of kombucha or black tea under different pH against *E. coli* and *S. aureus*.

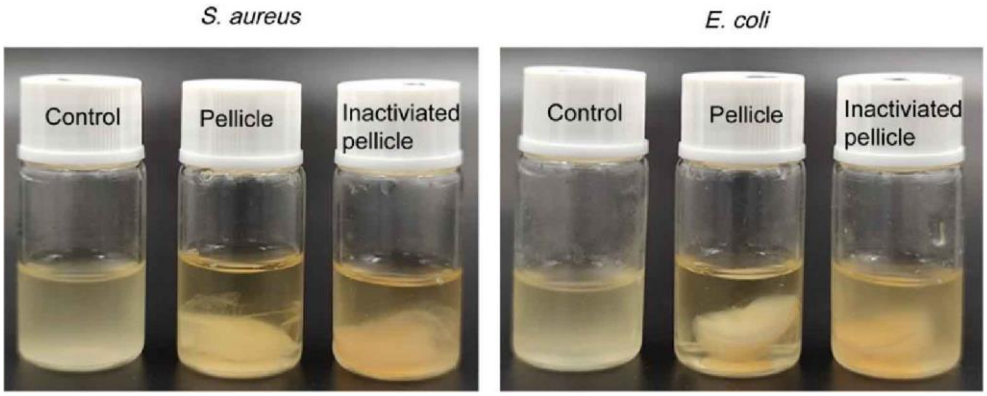

Figure S8. Digital photos of the *S. aureus* (5 mL) and *E. coli* (5 mL) solutions cocultured with the pellicles for 48 h.

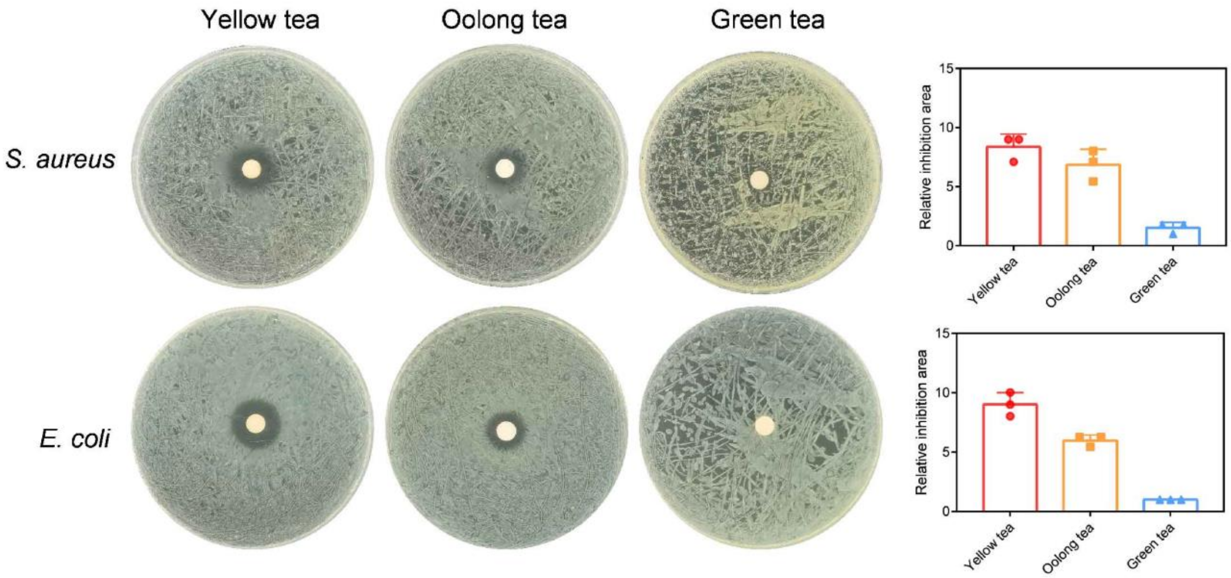

Figure S9. Inhibition zones surrounding the different tea-infused paper disks against *S. aureus* and *E. coli*.

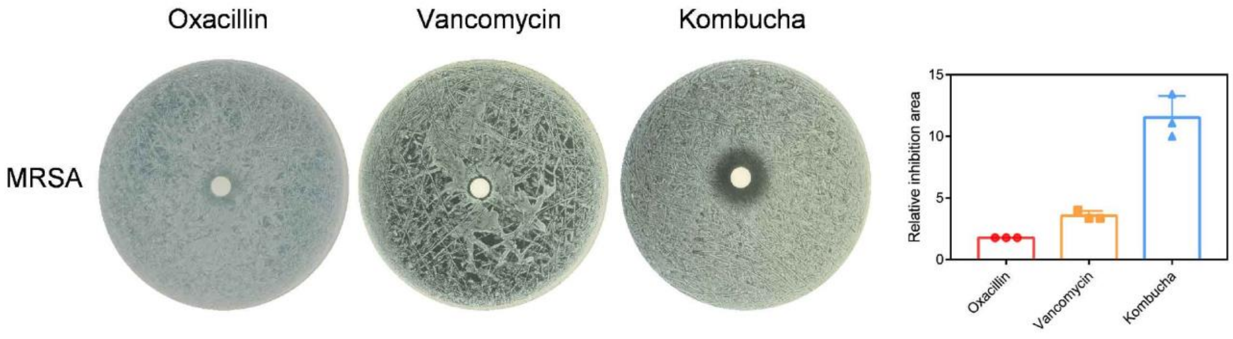

Figure S10. Inhibition zones surrounding the paper disks pretreated with oxacillin (1 μg/mL), vancomycin (10 μg/mL), or kombucha against MRSA.

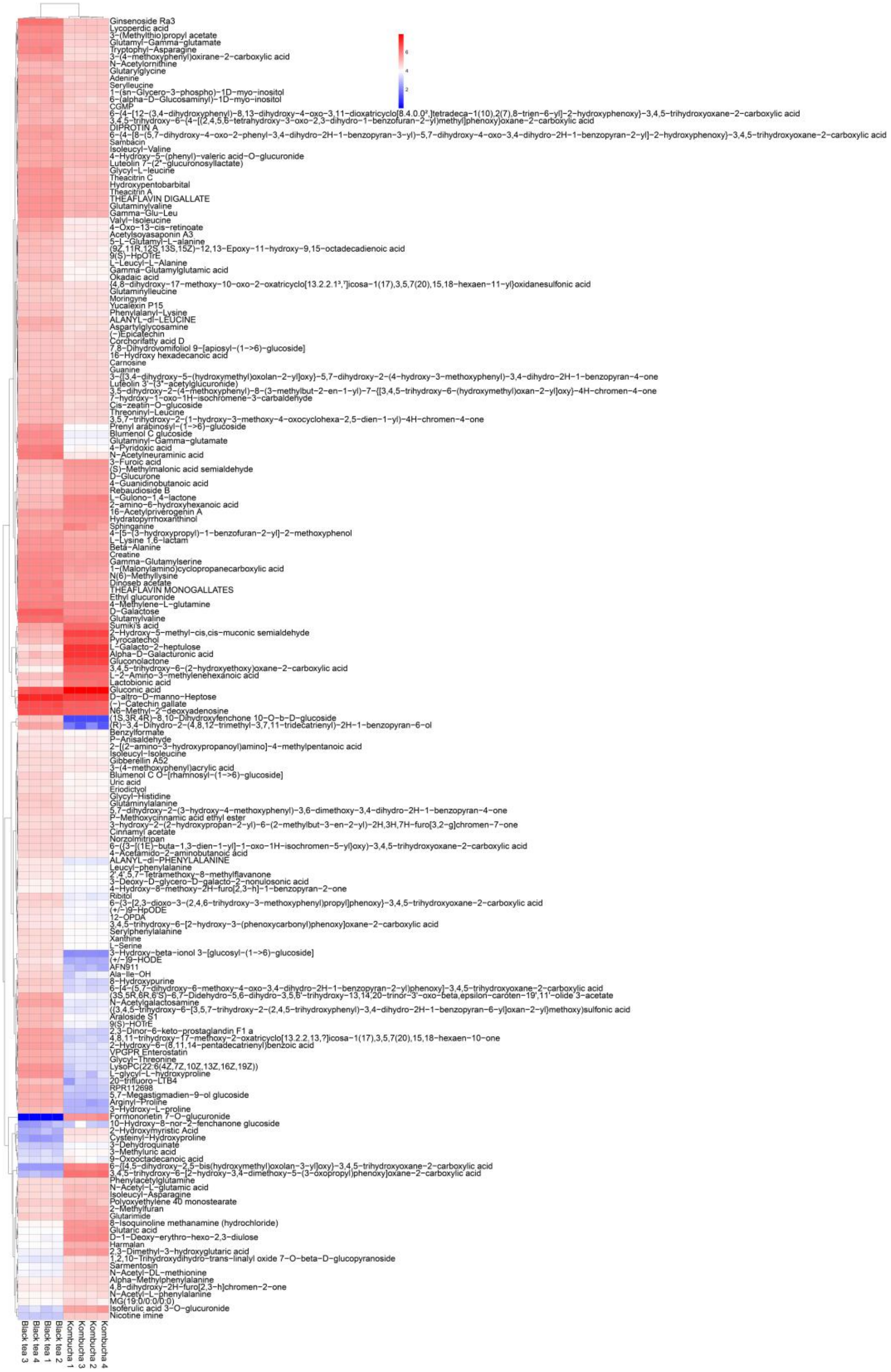

Figure S11. The changes in metabolites of the medium after kombucha fermentation.

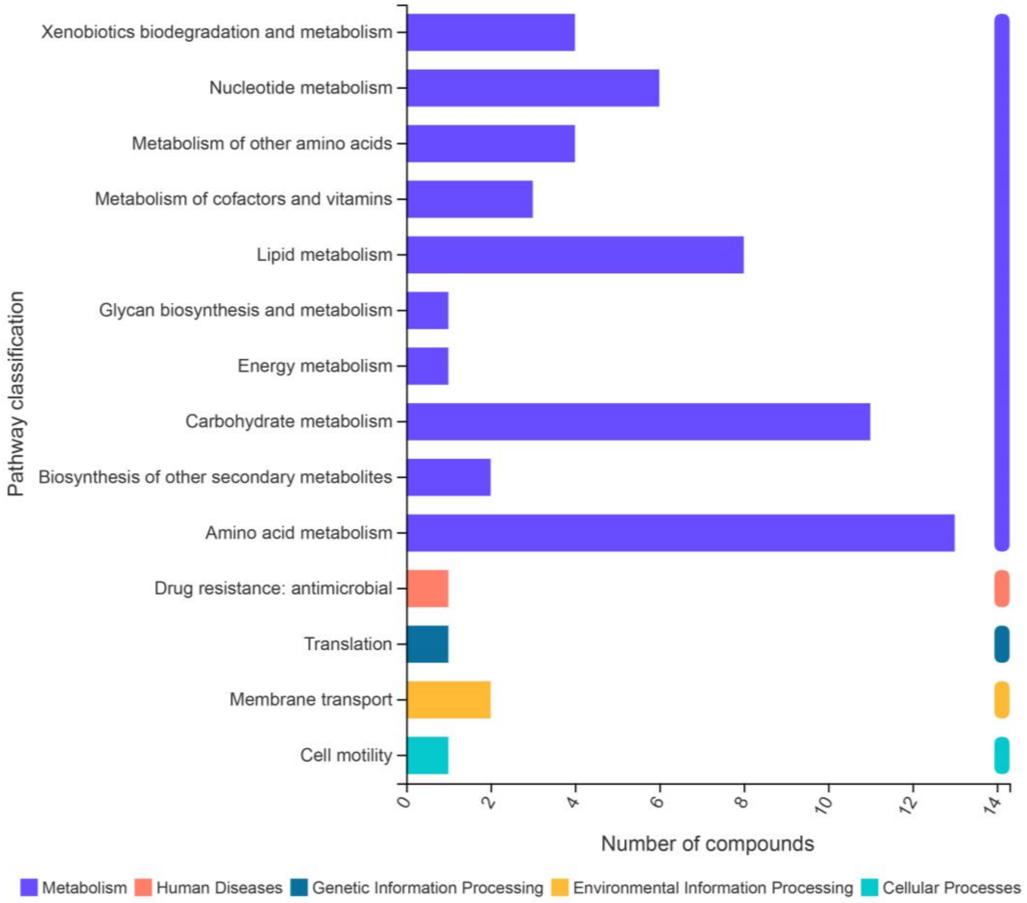

Figure S12. The KEGG metabolic pathways involved in compounds of the medium after kombucha fermentation.

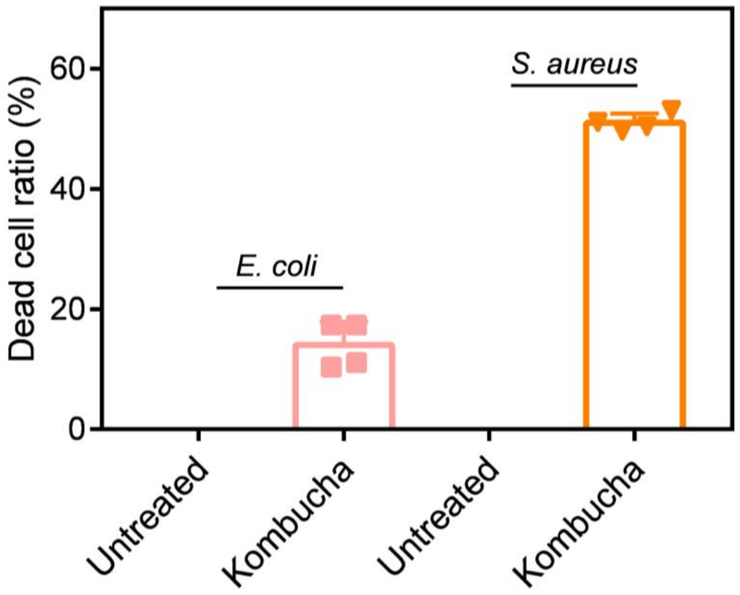

Figure S13. Quantification of the bacteria staining with MycoLight™ Green (green) and PI (red)**.** Each value represents the mean ± s.d. (n = 4).

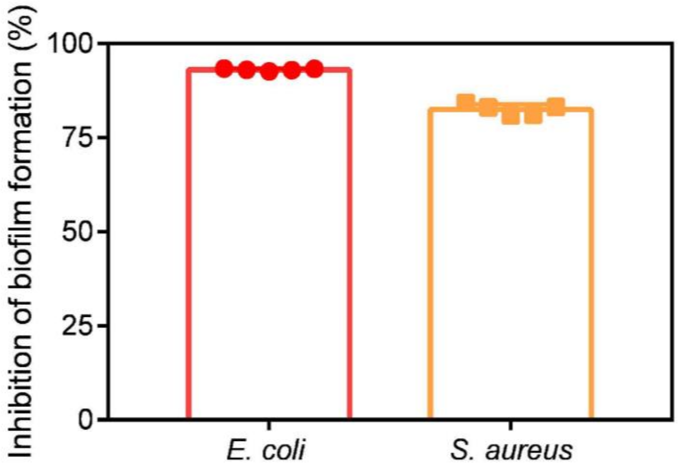

Figure S14. Inhibition percentage of biofilm formation of gel with kombucha against *S. aureus* and *E. coli*. Each value represents the mean ± s.d. (n = 5).

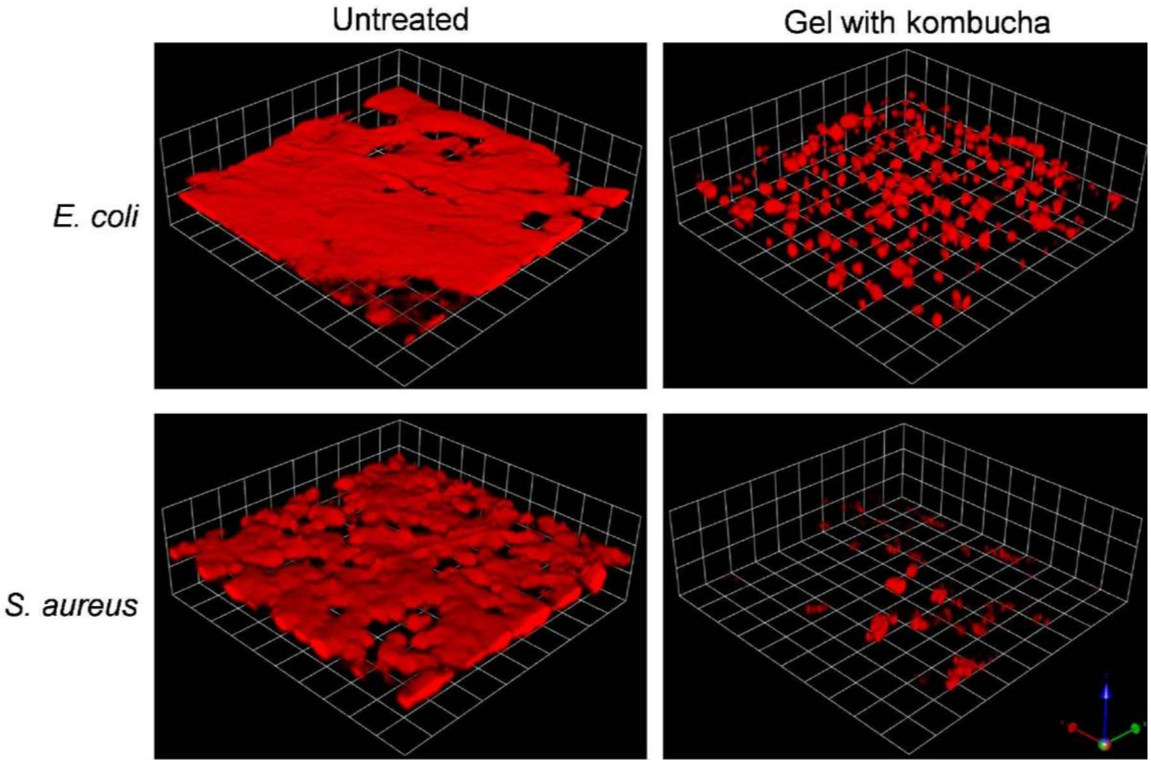

Figure S15. CLSM 3D images of *E. coli* and *S. aureus* biofilms after being treated by gel with kombucha.

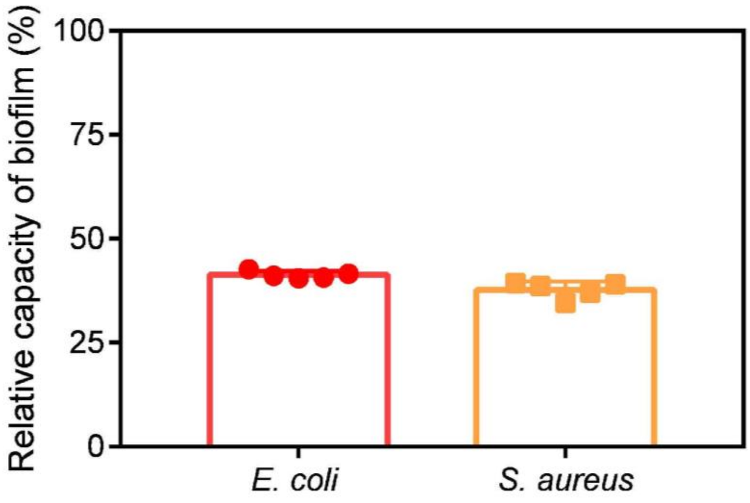

Figure S16. Disperse percentage of biofilms by gel with kombucha against *E. coli* and *S. aureus*. Each value represents the mean ± s.d. (n = 5).

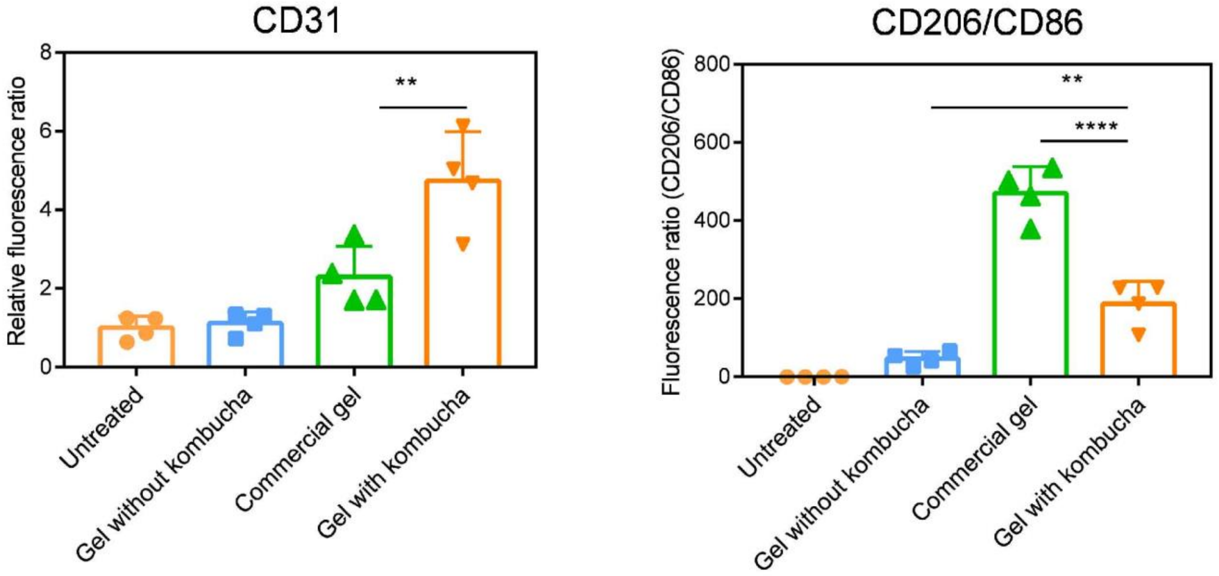

Figure S17. Quantification of the immunofluorescence images of CD31, CD206/CD86. Significantly different (one-way ANOVA): $^{**}P < 0.01$ and $^{****}P < 0.0001$.

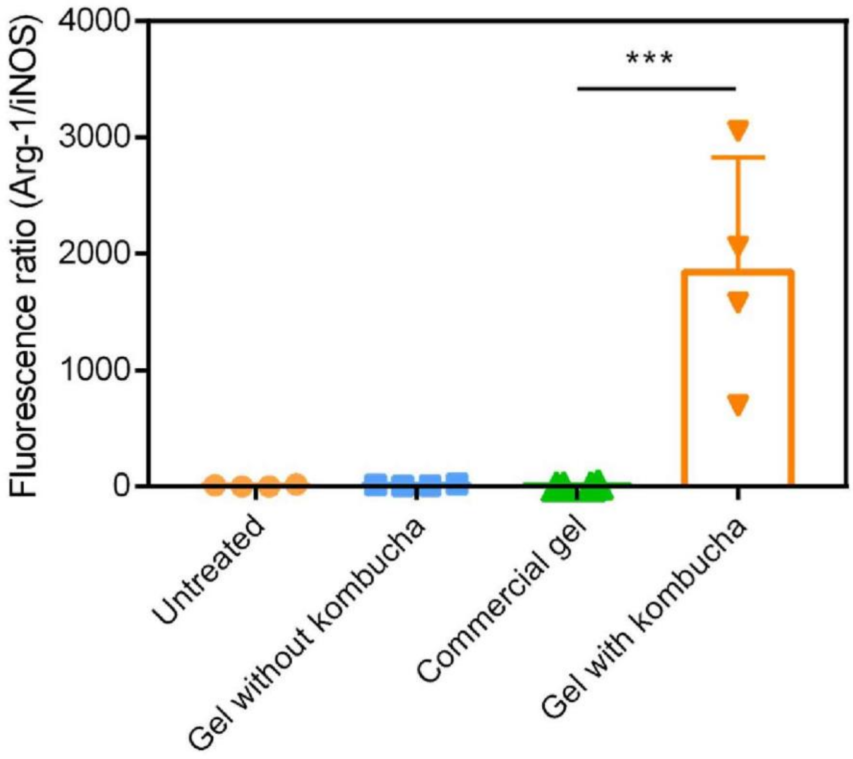

Figure S18. Quantification of the immunofluorescence images of Arg-1/iNOS. Significantly different (one-way ANOVA): ***$P < 0.001$.
